# Large scale extraction of poly(3-hydroxybutyrate) from *Ralstonia eutropha* H16 using sodium hypochlorite

**DOI:** 10.1186/2191-0855-2-59

**Published:** 2012-11-19

**Authors:** Daniel Heinrich, Mohamed H Madkour, Mansour A Al-Ghamdi, Ibraheem I Shabbaj, Alexander Steinbüchel

**Affiliations:** 1 Institut für Molekulare Mikrobiologie und Biotechnologie, Westfälische Wilhelms-Universität Münster, Corrensstraße 3, D-48149, Münster, Germany; 2 Environmental Sciences Department, Faculty of Meteorology, Environment and Arid Land Agriculture, King Abdulaziz University, Jeddah, Saudi-Arabia

**Keywords:** Poly (3-hydroxybutyrate), Polyhydroxyalkanoates, Ralstonia eutropha, PHA recovery, Sodium hypochlorite, Downstream process

## Abstract

Isolation of polyhydroxyalkanoates (PHAs) from bacterial cell matter is a critical step in order to achieve a profitable production of the polymer. Therefore, an extraction method must lead to a high recovery of a pure product at low costs. This study presents a simplified method for large scale poly(3-hydroxybutyrate), poly(3HB), extraction using sodium hypochlorite. Poly(3HB) was extracted from cells of *Ralstonia eutropha* H16 at almost 96% purity. At different extraction volumes, a maximum recovery rate of 91.32% was obtained. At the largest extraction volume of 50 L, poly(3HB) with an average purity of 93.32% ± 4.62% was extracted with a maximum recovery of 87.03% of the initial poly(3HB) content. This process is easy to handle and requires less efforts than previously described processes.

## Introduction

Today, plastic materials are part of humanities everyday life and are indispensible for numerous consumer goods and applications. Currently, they are mostly produced from fossil resources (Chanprateep 2010). In addition to persistent, non-biodegradable plastics, an increasing demand for biodegradable plastics exists (Poirier et al. 1995). As a potential substitute for conventional petrochemically produced plastics, biodegradable polymers (biopolymers) synthesized from renewable resources have attracted considerable interest in the past decades (Steinbüchel 2001). Among the different types of biopolymers, polyhydroxyalkanoates (PHAs) present a promising group of polymers, as they are biocompatible, thermoplastic and nontoxic, which makes them suitable for various applications in industry, medicine or agriculture (Anderson and Dawes 1990, Steinbüchel 2001, Van der Walle et al. 2001). Although PHAs with equivalent functionality to conventional plastics can be produced by bacterial fermentation at a large scale, their production accounts so far for only a small fraction of the global plastic production, which is mostly due to the comparably high production costs (Keshavarz and Roy 2010).

A major factor of the costs of PHA production is the recovery of the product from the cellular matter. Besides a reasonable purity and high recovery rate of the polymer, the use of inexpensive and environmentally friendly chemicals and the application of only few, simple separation steps are crucial in order to achieve an economically feasible process. Many studies have been done on PHA extraction, including the use of organic solvents (Ramsay et al. 1994) as well as chemical or enzymatic digestion of the major parts of the biomass (Berger et al. 1989, Kapritchkoff et al. 2006) including the proteins (phasins) which are tightly bound to the granules (Pötter and Steinbüchel 2005), or the mechanical disruption of the cells (Tamer et al. 1998). However, many of described extraction processes prove to be too costly, inefficient or elaborate when scaled up.

This study shows a simple method for high purity PHA extraction by the example of poly(3-hydroxybutyrate), poly(3HB), recovery from cells of *Ralstonia eutropha* H16 using sodium hypochlorite, which is suitable for use at industrial scale.

## Materials and methods

### Microorganism used in this study

The wild type *Ralstonia eutropha* strain H16 (DSM 428, Wilde 1962) was used in this study.

### Growth of cells

Cells of *R. eutropha* H16 were obtained by fed-batch fermentation at 30°C using a Biostat D650 (Sartorius) bioreactor at the 400 L-scale. Temperature, pH, foam, optical density and dissolved oxygen were measured by probes and sensors connected to the bioreactor. Concentrations of carbon dioxide and oxygen in the gas leaving the bioreactor were determined by the measuring devices Uras 10 E and Magnos 6 G *1* (both Hartmann & Braun AG). A pH of 7 was maintained by the addition of 4 M HCl and NaOH. A drop in the amount of dissolved oxygen was compensated by increasing the stirrer speed (100–400 rpm) or the airflow rate (100–400 L min^-1^). Foam was controlled by a mechanical foam destroyer or by the addition of a 50% (v/v) Struktol (Schill & Seilacher) solution.

Mineral salt medium with the following composition was used: 2.0 g L^-1^ (NH_4_)_2_HPO_4_, 2.1 g L^-1^ KH_2_PO_4_, 0.2 g L^-1^ MgSO_4_ × 7 H_2_O, 0.1 g L^-1^ CaCl_2_ × 2 H_2_O, 0.006 g L^-1^ FeCl_3_ × 6 H_2_O, 0.1 ml L^-1^ of trace element solution SL6 (Schlegel et al. 1961) and 30 g L^-1^ of sodium gluconate as carbon source. During cultivation, a carbon/magnesium solution (400.0 g L^-1^ sodium gluconate, 5 g L^-1^ MgSO_4_ × 7 H_2_O), an ammonia solution (57 g L^-1^ (NH_4_)_2_HPO_4_, 257 g L^-1^ NH_4_Cl) and a trace element solution SL6 were fed to gain a high cell density. About 18 h before the cells were harvested, the cells were cultivated under nitrogen-limitation with an excess of carbon in the medium in order to stimulate PHA accumulation.

### Cell harvest and preparation of cells for extraction

After separation from the culture broth with a CEPA type Z61 continuous centrifuge (Carl Padberg Zentrifugenbau GmbH), the biomass was frozen at −30°C and lyophilized. After lyophilization, the dried cells were grinded to a powder with a conventional blender.

### Extraction of poly(3HB)

The extraction of poly(3HB) from cells of *R. eutropha* H16 was carried out in different volumes, ranging from 0.1 L glass bottles to steel barrels with a volume of up to 50 L. For this dry and pulverized cells (30 g/L) were suspended in an aqueous 13% (v/v) sodium hypochlorite solution with a pH of 12.3 and incubated at room temperature for 1 h. As cell lysis with hypochlorite is strongly exergonic and generates a severe amount of foam, in- or external cooling was applied, depending on the total volume of the vessel to prevent a strong temperature increase. In order to accelerate the sedimentation of extracted PHB, half of the initial volume of water was added to the solution, which was then incubated for at least 8 h at room temperature. After removing the supernatant, the polymer was washed at least twice with water and once with isopropanol (15 min, 4,000 × g, 4°C). Each washing step required 1 L of the respective liquid. The purified polymer was dried by lyophilisation.

For comparison, poly(3HB) was isolated from lyophilized cells with hot chloroform in a Soxhlet extractor. The extract was then concentrated through rotary evaporation and the polymer was precipitated in 10 vol of ice-cold methanol. Upon separation of the poly(3HB) from methanol through centrifugation (15 min, 4,000 × g, 4°C), the polymer was left to dry.

### Determination of poly(3HB)

Quantitative and qualitive analysis of cells and the isolated polymer for poly(3HB) content and composition were done by gas chromatography (GC). For this, cell samples or samples of isolated poly(3HB) were subjected to methanolysis in presence of hydrosulfuric acid as described previously (Brandl et al. 1988, Timm et al. 1990).

### Molecular weight determination of poly(3HB)

Molecular weights of samples of isolated poly(3HB) were determined through Gel Permeation Chromatography (GPC) analysis using a Varian GPC50 with an RI detector. Samples were separated in two 30 cm PLGel Mixed C columns (Agilent) at 30°C with distilled chloroform as the mobile phase. A standard curve was prepared with polystyrene samples of narrow polydispersity. Poly(3HB) samples were prepared by dissolving in chloroform with stirring for 3 h at room temperature. Molecular weights were determined by the retention times on the column with injection intervals of 30 min. Poly(3HB) (Sigma) of known molecular weight was used as a control.

## Results

**Figure 1:**
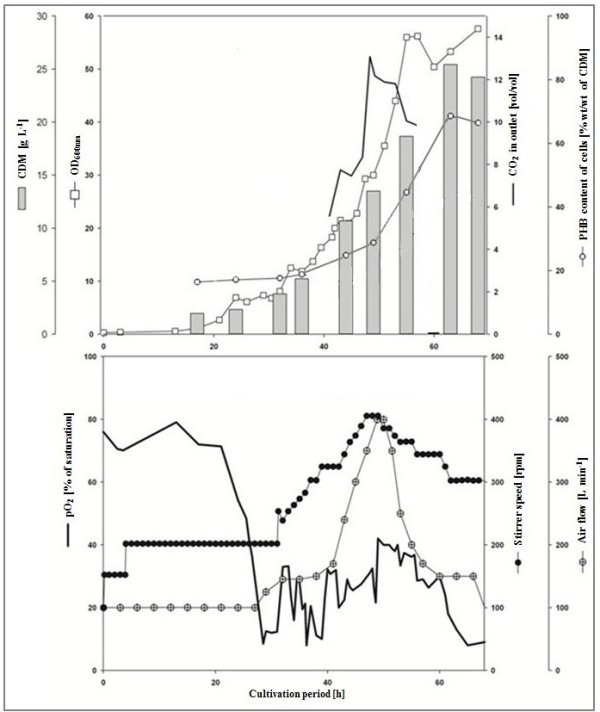
**Fed-batch cultivation of *R. eutropha *H16 in a Biostat D-650 stirred tank reactor.** The bioreactor contained 400 L of mineral salt medium with sodium gluconate as carbon source. A 20 L preculture was grown in the same medium. Cells were grown for 68 h at 30°C and harvested by continuous centrifugation. Due to technical problems, the concentration of carbon dioxide leaving the bioreactor could only be measured between the 40th and 58th hour.

Fed-batch cultivation of *R. eutropha* H16 (Figure 1) was carried out in mineral salt medium with an initial content of 30 g L^-1^of sodium gluconate and 2.0 g L^-1^ of (NH_4_)_2_HPO_4_ . A pH of 7 was automatically maintained throughout the process by adding 4 M HCl or NaOH. A total yield of 10.8 kg of dried cell mass was obtained through a single fermentation of *R. eutropha* H16 at the 400 L scale with subsequent harvesting of the cells with a continuous centrifuge. The poly(3HB) content of the cells was 65.2% ± 1.6% (w/w), as measured by GC analysis. The cell density at the point of harvest was 24.2 g L^-1^ and the productivity regarding accumulated poly(3HB) for the period of the main fermentation was 0.23 g L^-1^ h^-1^. At various time points during fermentation, samples of cells were stained with the hydrophobic dye nile red. The stained poly(3HB) granules were examined with a fluorescent microscope. Upon nitrogen limitation of the culture after 40 h of cultivation cells increasingly displayed large granules of poly(3HB).

In order to determine the maximum amount of cell mass that could be efficiently digested by a 13% sodium hypochlorite solution, small-scale extraction experiments were carried out at first. For this, different amounts of dried cell mass ranging from 1 – 5 g were suspended in 100 ml of a 13% (v/v) sodium hypochlorite solution. At a biomass concentration higher than 30 g L^-1^ (w/v), the sodium hypochlorite solution was saturated, which was indicated by non-digested biomass remaining on top of the supernatant after sedimentation of the extracted polymer. Therefore, all subsequent extractions were carried out at a biomass concentration of 30 g L^-1^ (w/v).

After digestion of the non-poly(3-HB) biomass, the polymer sedimented to the ground. However, sedimentation proceeded slowly, and the polymer did not sediment completely. This problem could be overcome by the addition of water. The much faster sedimentation of the polymer was accompanied by a clearer separation. Consequently, the sedimented poly(3HB) could be decanted from the supernatant much easier. About 90% of the supernatant could be removed by decanting, so that 10% of the initial volume was left to be centrifuged.

**Figure 2:**
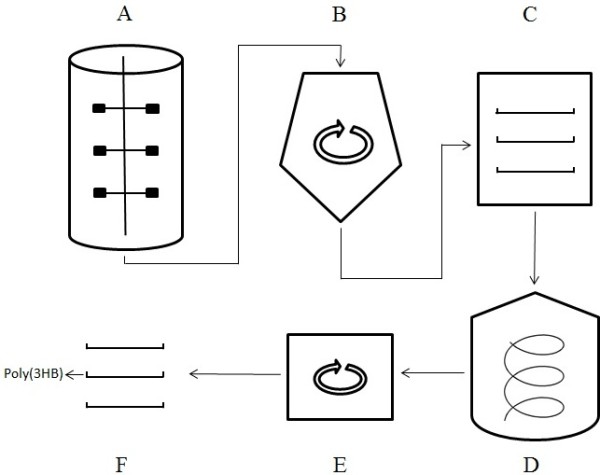
**Schematic diagram displaying the process for poly(3HB) production.** (**A**) Cultivation of *R. eutropha* H16. (**B**) Separation of bacterial cells from the culture broth by continuous centrifugation. (**C**) Freeze drying. (**D**) Digestion of non-poly(3HB) cell matter by sodium hypochlorite with internal cooling and separation of the polymer through sedimentation. (**E**) Washing of the polymer with isopropanol with a small scale centrifuge. (**F**) Drying of the polymer through evaporation of isopropanol.

After washing the isolated poly(3HB) three times with water, no odour of hypochlorite was present in the product. The extracted and purified polymer appeared as white powder exhibiting a purity of 95.66% ± 1.31% (w/w). About 91.32% ± 2.14% of the poly(3HB) initially present in the dried cells was recovered at the 0.1 L scale. With an increase in capacity to the 50 L scale (Figure 2), the purity of the isolated poly(3-HB) did not decrease significantly, and an average purity of 93.32% ± 4.62%. (w/w) was achieved as measured in a total number of seven batches (Table 1). The maximum recovery that could be achieved with regard to the initial concentration of poly(3HB) in the cells was 87.03%. It was very crucial to start the hypochlorite treatment with well pulverized cells; otherwise the recovery decreased significantly.

**Table 1:** **Overview of poly (3HB) recovery from cells of *R. eutropha *H16**

**Batch no.**	**Recovered poly(3HB) [% of initial content]**	**Purity of recovered poly(3HB) [%]**	**Molecular weight (Da) of recovered poly(3HB)**
1	43.14	94.76	459733
2	47.03	89.32	682630
3	41.69	98.63	not determined
4	69.06	99.42	769389
5	46.44	91.40	530447
6	62.19	92.79	832705
7	87.03	86.94	489320

Between 750- and 820 g of dried cells were digested in a 13% (v/v) sodium hypochlorite solution at a concentration of 30 g L^-1^ at pH 12.3. All batches were parts of the cells obtained by fermentation at the 400 L scale.

GPC analysis of the poly(3HB) extracted with sodium hypochlorite revealed molecular weights between 460,000 and 830,000 Da with a polydispersity index (PDI) of 2.45 on average. In contrast poly(3HB), which was extracted with chloroform from the same cells, displayed a molecular weight of 1,700,000 kDa with a PDI of 1.67.

## Discussion

Through fermentation of *R. eutropha* H16, a sufficient amount of biomass for a large scale extraction experiment was produced. However, the productivity regarding accumulated poly(3HB) was below average, which was due to the long lag phase of the culture. The aim of this study, was to develop a simple and efficient downstream process for the production of poly(3HB). The most common methods for PHA recovery from bacterial cells involve the use of (halogenated) solvents (Ramsay et al. 1994, Elbahloul and Steinbüchel 2009). After the solvent modifies the cell membrane and dissolves PHA, separation of the polymer from the solvent is necessary. This can either be mediated by evaporation of the solvent, or precipitation of PHA by a non-solvent, such as ethanol, methanol or even water (Zinn et al. 2003, Hänggi 1990). Although extractions involving the use of solvents have accomplished purities of higher than 98% and recoveries of more than 95% (Zinn et al. 2003, Lafferty and Heinzle 1978), a complex setup for the execution of successive steps is required.

In contrast, PHA separates from lysed cell matter in an aqueous sodium hypochlorite solution through sedimentation because it is insoluble in water. Furthermore, sodium hypochlorite is not volatile or combustible and requires therefore less safety measures than most solvents. As studies on PHA recovery using sodium hypochlorite suggested a separation of the polymer by centrifugation (Berger et al. 1989, Hahn et al. 1994, Ribera et al. 2001) one has to consider the total volume of a large-scale extraction process. Since the maximum concentration of cell matter that can be digested in a sodium hypochlorite solution is much lower than in solvents, separation through centrifugation would exceed available capacities. Therefore, we show that digestion of cell matter using a relatively high concentrated sodium hypochlorite solution with the subsequent addition of water mediates efficient digestion of non-PHA biomass and clear separation, which is due to the insolubility of PHAs in water. Thereby, the volume to be centrifuged is reduced to a minimum. A sodium hypochlorite concentration of 13% is well suited for the process, as it is commercially available and efficiently digests non-PHA biomass without generating excessive, non-manageable heat, the use of a higher concentrated solution would lead to. Additional steps such as solving the polymer in chloroform with a subsequently required precipitation (Hahn et al. 1994, Sayyed et al. 2009) are not necessary in order to achieve a reasonable purity and product recovery. Alternatively, the polymer can be simply washed with e. g. isopropanol to remove remaining lipids. In addition, we observed that instead of an increase of the temperature to 30°C or 37°C (Hahn et al. 1994, Ribera et al. 2001), in- or external cooling is required since the digestion of biomass using sodium hypochlorite is strongly exothermic.

In contrast to previous studies, our protocol applies a biomass to hypochlorite ratio ideal for a large scale extraction of poly(3HB). The concentration of dried *R. eutropha* H16 cells, which are digested in a sodium hypochlorite solution is three-fold higher than the concentration of 1% (w/v) used by Berger et al. ( 1989), thereby reducing the total volume as well as the amount of chemicals and energy required for the extraction process considerably. Whereas Hahn et al. ( 1994) suggested a biomass concentration of 4% (w/v) to be digested in a 30% (v/v) sodium hypochlorite solution, we showed that a lower sodium hypochlorite concentration is sufficient to achieve a complete digestion of an only slightly lower concentration of non-poly(3HB) biomass. A lower hypochlorite concentration is advantageous in order to reduce the temperature of the process and also to limit the degradation of the polymer (Berger et al. 1989).

In addition, our study also showed that it is inevitable for the extraction process to grind lyophilized cell matter thoroughly in order to present a maximum surface area and thereby provide the basis for the complete digestion of the non-PHA biomass. Of the seven large scale batches, only the last batch, where pulverized dried cell matter from the bottom of the container was extracted, resulted in a reasonably high recovery rate. In contrast, batches with chunks of cell matter displayed less surface area to the hypochlorite solution, so that much of the cell matter remained undigested. Therefore, only the recovery rate, but not the purity of the isolated polymer varied in the seven different batches. In order to confirm this, further extraction experiments with pulverized cell matter could be carried out.

Assuming chloroform extraction does not lead to degradation of poly(3HB), GPC analyses revealed a decrease of the original molecular weight of poly(3HB) isolated through treatment with sodium hypochlorite by 50 – 70%. Additionally, the isolation with sodium hypochlorite led to a polymer of higher dispersity. As most applications demand polymers of high molecular weights and low dispersities, the polymer degradation through treatment with hypochlorite is a drawback of the process. However, our protocol does not lead to a more severe degradation than in previous studies, where poly(3HB) caused a decrease in molecular weight of up to 50% (Hahn et al. 1995) and even 80% (Berger et al. 1989) when extracted with sodium hypochlorite at comparable parameters.

In summary, we developed an ideal method for large scale isolation of poly(3HB) from dried cell mass, which (i) recovers a product of high purity with little turnover, (ii) proves to be simple and cost-saving by mediating efficient separation of the polymer from the lysed cells without the use of additional chemicals or a large scale centrifuge, (iii) limits the total volume of the extraction process with an optimized ratio of biomass to hypochlorite creating a manageable amount of heat and foam and (iv) presents technical solutions to overcome challenges that emerge with the upscale of an extraction process with sodium hypochlorite.

## Competing interests

The authors declare that they have no competing interests.
